# Supplementary material for: Methylated cis-regulatory elements mediate KLF4-dependent gene transactivation and cell migration
Source: eLife. 2017 May 29;6:e20068. doi: 10.7554/eLife.20068 (PMC5466421; doi:10.7554/eLife.20068)
Supplement: Supplementary file 2. — DOI: http://dx.doi.org/10.7554/eLife.20068.020 [file elife-20068-supp2.docx]

**Table 2: Primer sequences of bisulfite sequence**

| gene | primer (left) 5’-3’ | primer (right) 5’-3’ |  |  |
| --- | --- | --- | --- | --- |
| CTH | TTTTGGGTTTAAGTGGTGTTAGTTT | AAACAACTATTTTCTTAACCACCCTC | | |
| DHDDS | GTGGTTTTTTGATTATTTTTTTAGT | ACAAAATCCCCTCAATTTAAATAAC | | |
| ESYT2 | GTTTTTGGGAGTTGTTGGTAAA | CACACTAACTAAACAACCTCACACC | | |
| FAM65A | TTTTTAGGAGAAAGGAGTTAGGAAG | AACCATCTCCAAACTAACCTCTACA | | |
| IDH1 | TTTAGTTAGGTGGTTTTGGGAAAG | ACCTTATCATTTATTTCATTATAAAATAAA | | |
| LGALS3 | TGGAGGGTAGAGTATAGGTTGTG | AAAAAAAACTAACTAAACAAAAAC | | |
| LMO7 | AATAATTTTTTATTAAGGGTTTTTT | AACCTACATATCCCAATATAAACTC | | |
| MIDN | TTGGTTTTAATTTTTTGATTTTGTG | CACCTTAACAAACACACCCAAC | | |
| MIR937 | TATTGTAGGTTGGGTTGGGTAGA | CCTCTAAAACTCATAAAACCATCCA | | |
| PHLDB2 | TGTAATTTTAGTATTTTTGGGAGGT | TACAATCTTAACTCATTACAACCCC | | |
| PKM2 | GTTTAGGTTGGAGTGTAGTGG | TCAATTATTCTACTTTTAAAAAAATTATCT | | |
| RABGEF1 | GTTTAGGTTTTTTTTGATGGTGATAG | CAAACTAATCTCAAACTCCTAACCTC | | |
| RAC1 | TTAATTAAAGTGTTGGGATGATAGA | AAAAAAATCTCTTAAACCTAAAAAAC | | |
| RHOC  S100A4 | AAAGGGGATGGGTATATTTTTTTTA  ATATTTAGTTTTGGGAGGGAAAAGA | ACCTAAAATTTCCAAAAATCCAATT  CCTAAAAAAACCCAAAACAATAAAC | | |
| UGDH | TTTTATTATGTTAGTTAGGATGGTTT | ATTTAATTTATTATAACTCTCCCAAAATAC | | |
